# Behavior, hormone, and gut microbiota change by YYNS intervention in an OVX mouse model

**DOI:** 10.3389/fcimb.2024.1445741

**Published:** 2024-11-07

**Authors:** Huajuan Lei, Jian Liu, Juan Deng, Pan Zou, Zixiang Zou, Ziou Li, Honghui Li, Lin Luo, Zhoujin Tan

**Affiliations:** ^1^ Department of Anesthesiology, the First Hospital of Hunan University of Chinese Medicine, Changsha, China; ^2^ Department of Chinese Medicine, ChangSha Medical University, Changsha, China; ^3^ Department of Innovation Experiments Center, the First Hospital of Hunan University of Chinese Medicine, Changsha, China; ^4^ Department of Anesthesiology, Changsha Hospital Affiliated with Hunan University of Chinese Medicine, Changsha, China; ^5^ Department of Radiology, the First Hospital of Hunan University of Chinese Medicine, Changsha, China; ^6^ Department of Gynaecology, the First Hospital of Hunan University of Chinese Medicine, Changsha, China; ^7^ Department of Radiology, Changsha Hospital Affiliated with Hunan University of Chinese Medicine, Changsha, China; ^8^ Department of Orthopedics, the First Hospital of Hunan University of Chinese Medicine, Changsha, China; ^9^ Department of Medicine, Hunan University of Chinese Medicine, Changsha, China

**Keywords:** Yangyin-ningshen formula, premenopause depression, gut flora, endocrine-brain-gut-microbiota axis, OVX, E2-gut-brain axis

## Abstract

**Object:**

Perimenopause depression disorder (PDD) is a very common problem in clinical practice and is characterized by depression and autonomic nervous symptoms, including hot flashes, palpitation, and night sweating. In addition, the comorbidity of menopause depression has long been an integral component of the estradiol (E2) shortage. Previous studies have suggested that the mechanisms underlying this comorbidity involved overlap of endocrine and cerebellar networks. Emerging evidence has shown that the endocrine–brain–gut–microbiota axis plays a key role in the regulation of affective disorders. Yangyin-ningshen formula (YYNS) is a traditional Chinese decoction tailored by Yijintang for menopausal depression intervention. Thus, we hypothesized that the YYNS may be involved in the menopause depression alleviation through the endocrine–brain–gut–microbiota axis.

**Methods:**

To verify this, we constructed a bilateral ovariectomy (OVX) mouse model to simulate menopausal-related depression. Subsequently, behavioral tests including the open field test (OFT) and the forced swimming test (FST) were conducted to examine the depression state post-OVX. With YYNS or E2 intervention, enzyme-linked immunosorbent assay (ELISA) was used to determine the serum sex hormones level. 16S rRNA gene sequencing and liquid chromatography-mass spectrometry (LC-MS) were used to analyze the microbiome of the colon samples collected from mice in the sham surgery group (CSH), the OVX model group (CMD), the OVX with E2 hormone intervention group (CHM), and the OVX with YYNS intervention group (CYYNS). One week after OVX, CMD, CHM, and CYYNS showed depression in OFT, FST. Three weeks post-OVX, CHM and CYYNS showed a notable relief of depression; CMD shaped the OTUs shrinkage; and OTUs were raised in the sham, CHM, and CYYNS group. The CMD group showed that the abundance of Actinobiota decreased but that of Bacteriodia increased. The relative abundance of the genus varied in each group. Moreover, functional correlation of changes in sex hormone and gut microbes between different groups showed that the PRL level was negatively correlated with *Odoribacter*. T level was positively correlated with *Lachnospiraceae NK4A136 group* and *Odoribacter* abundance (*p* < 0.05).

**Conclusion:**

Our results not only offer novel insights into the sex hormones and depression with OVX mice but also build an important basis for E2 or YYNS therapeutic efficacy on PDD, which provide for future research on this etiology through the endocrine–brain–gut–microbiota network.

## 1 Introduction

Perimenopausal depressive disorder (PDD) is primarily characterized by depressive and anxiety-related symptoms. Some patients even present major depression due to insufficient estrogen supply caused by the decline in ovarian function (Bromberger et al., 2011; Freeman et al., 2006). PDD is most prevalent in women aged 45–50 years, coinciding with the menopausal transition period (Tang et al., 2019). Currently, the average age of 88% of women in perimenopause is 51.4 years old, and the prevalence of menopausal depression in China in 2009 was 36.3%, with 18.6% of mild depression, 15.3% of moderate, and severe depression of 3.7% (Zeng et al., 2019), and it has a tendency to rise year by year. PDD not only affects women’s physical and mental health but also poses a great healthcare and economic burden to the family and society. PDD is closely related to changes in sex hormone, especially estrogen levels. In clinical practice, estrogen replacement therapy and antidepressants are widely applied for PDD treatment, but the risk of breast and endometrial cancer has elevate greatly in women with long-term hormone exposure (Marjoribanks et al., 2017).

YYNS (Yangyin-ningshen formula, YYNS) comprises key ingredients such as Rehmannia glutinosa 15 g, Parched Semen Ziziphi Spinosae 15 g, Paeoniae Radix Alba 10 g, Coastal Glehnia Root 10 g, Moutan Cortex 10 g, *Angelicae sinensis* Radix 8 g, Corni Fructus 12 g, and Bupleuri Radix 12 g. These components synergistically provide yin-nourishing, blood-replenishing, liver-soothing, and antidepressant effects, which can modulate the endocrine system and mitigate depressive symptoms associated with estrogen fluctuations. YYNS has been extensively utilized to alleviate depressive symptoms linked to the decline in estrogen levels during the perimenopausal period. However, the precise mechanisms underlying its anti-PDD effects remain to be fully elucidated. Hormone level alteration is the most significant change during menopause transition including PRL and E2 concentration (Burger et al., 2008). PRL is a critical hormone involved in regulating lactation, reproduction, and immune responses. Changes in PRL levels have not been associated with mood disorders, but it is known to interact with estrogen pathways to influence depression due to its effects on dopaminergic pathways and hypothalamic–pituitary–ovarian axis (HPO) modulation (Jiao et al., 2023). Therefore, measuring PRL is crucial for understanding the interplay between estrogen and prolactin in the neuroendocrine mechanisms underlying PDD. E2 plays a key role in the neurobiology of aging, in part due to extensive inter-connectivity of the neural and endocrine system (Jett et al., 2022). E2 is the most potential bio-formation within the nervous system (Brann et al., 2022). The main resource of cerebral E2 is located on dendrite and axonal aromatase (ARO) in neurons, where E2 is synthesized via testosterone (T) or cholesterol (Lu et al., 2019). Both T and E2, two key hormones in the endocrine system, are derived from a common precursor: cholesterol. Cholesterol, a sterol lipid found in the cell membranes of animals, serves as a vital building block for the biosynthesis of these hormones in the periphery. However, the source of estrogen within the mammalian brain differs from that in the periphery. After crossing the blood–brain barrier, testosterone in the brain is converted into E2 (Azcoitia et al., 2021). Astrocytes can produce E2 only when neuronal injury occurs, but microglia and dendritic spine cells are unable to yield E2 in the brain (Brann et al., 2022). Abrupt fluctuations of estrogen levels are the main cause of menopause vagal motor symptoms such as flashes and palpitations; besides, a decrease in estrogen levels is an independent factor in triggering PDD (Burger et al., 2008). Cerebral 17β-E2 regulates cell growth, proliferation, and survival through participating in neuron regeneration by estrogen receptor alpha (ERα), brain-derived neurotrophic factor (BDNF), and postsynaptic density protein 95 (PSD 95) expression, especially in the hippocampus (Deng et al., 2022). In addition to neurons modulation potency, estrogen is also involved in the regulation of gut flora (Maeng and Beumer, 2023).

E2 serves as an independent factor in the psychophysiology process of PDD. The drastic changes in sex hormones level during menopause transition and the chronic stimulation of low hormone level can lead to the occurrence of PDD, which is beyond the factor of depression family history (Freeman et al., 2006), Besides cerebral behavior change triggered by low E2, there is also gut flora alteration. Recently, the relationship between PDD and gut flora is currently unknown. Gut flora already plays an important role in chronic diseases such as obesity (Kaliannan et al., 2018), diabetes (Abuqwider et al., 2023), dementia, Parkinson’s disease (Sasso et al., 2023), and osteoporosis (Guan et al., 2023). The gut microbiota may also be influenced by brain function through the “brain–gut” axis (Sasso et al., 2023), and current research has found that flora plays a vital role in the development of depression Parker et al., 2020). Vice versa, the disorder can also affect the function of neurons in the brain (Peters et al., 2022). With regard the specific traits of menopause transition and sex hormone importance, the tri-direction cross-talk between sex hormones, brain behavior changes, and gut flora needs further study as a whole, which refers to “E2–brain–gut–microbiota axis.”

Since the endocrine–brain–gut–microbiota axis is involved in the regulation of the brain activity, the overlap of endocrine, neural behavior, and gut flora network cannot be isolated from the mechanism of perimenopause depression. YYNS is a traditional Chinese decoction, which is tailored by Yijintang with menopause syndrome intervention. In recent years, studies have found that YYNS has a close relationship with gut microbiota. The formula can regulate the balance of the gut micro-ecosystem, reduce inflammation and oxidative stress, improve symptoms of anxiety and depression, and promote the production of short-chain fatty acids. However, whether sex hormone elicits mood dysfunction and gut microbiome alteration is poorly understood and should be investigated. Thus, we hypothesized that the YYNS and E2 may be involved in the menopause depression alleviation by modulating the endocrine–brain–gut–microbiota axis. This is a pilot study in which we used a multiomic approach for the first time to investigate the trio-directional effect of YYNS on the PDD, sex hormone, and gut flora and metabolic profiles in a OVX mouse model.

## 2 Materials and methods

### 2.1 Animals

A total of 24 SPF-grade C 57BL/6J female mice (20–25 g), 24 weeks old, were purchased from Hunan Slake Jinda SCXK (Xiang) Co. Ltd., no. 2019-0004. The mice were kept in the SPF-grade Laboratory Animal Center of the First Affiliated Hospital of Hunan University of Traditional Chinese Medicine at 22°CC–26°CC, under a 12-h light/dark cycle. All animal procedures were performed in accordance with the experimental protocol approved by the animal ethics committee of the First Affiliated Hospital of Traditional Chinese Medicine of Hunan University (The Ethical Review No. ZYFY20190418).

### 2.2 Experiment protocol

Animals were allowed to acclimatize for a week and randomly divided into CSH, CMD, CHM, and CYYNS groups, with six female mice in each group, respectively. Mice in each group were anesthetized with sodium pentobarbital intraperitoneally injected (50 mg/kg, i.p.) (Egashira et al., 2021). The CSH, CHM, and CYYNS group underwent an OVX procedure. The main steps were as follows: an incision was made 1 cm above the anterior superior iliac spine, the skin, muscle, and peritoneum, and then, both ovaries were resected (Liang et al., 2019). The CSH group only had the skin, muscle, and peritoneum incision and suture but no OVX. After surgery, the skin wound was disinfected with complex iodine once a day, and tetracycline ointment was applied locally once a day for a total of 10 days. To avoid impacting the intestinal flora, no oral or intravenous antibiotics were used. CSH and CMD groups were treated with sterile drinking water. CHM and CYYNS received E2 valerate 0.13 mg/(kg.d) (Tretinoin, Lot No. J 20171038, Bayer AG, Germany) and YYNS 21.5775 g/(kg.d). All mice had gavage once a day for 21 consecutive days. YYNS formula preparation was as follows: Rehmanniae Radix 15 g, Parched Semen Ziziphi Spinosae 15 g, Paeoniae Radix Alba 10 g, Coastal Glehnia Root 10 g, Moutan Cortex 10 g, Angelicae Sinensis Radix 8 g, Corni Fructus 12 g, and Bupleuri Radix12 g. Based on a dosage of 1.75 g/kg for a 60-kg adult, calculated according to body surface area, and using the formula for converting between human and mouse body weight (Db = Da·Rab), where the adult dosage is 1.75 g/kg and Rab is 12.33, the corresponding dosage of the YYNS for mice is calculated to be 21.5775 g/kg. The oral administration dosage was determined based on the mouse’s body volume (10 mL/kg), with gavage administered twice daily for 21 consecutive day. Behavior tests were taken before surgery and 1 week and three weeks postoperative. After behavior tests been completed, the mice were euthanized with sodium pentobarbital (60 mg/kg, i.p.) (Yu et al., 2022). Serum was collected and centrifuged in 5,000 rpm for 5 min and stored at −80°C. The colon of each mice were removed by sterile surgical instruments, the colon from the ileocecal region to the right hemicolon was selected, and the colon samples of each mice were freshly collected in an Eppendorf tube (EP) and stored at −80°C until further analysis.

### 2.3 Behavior evaluation

To assess the state of depression, mouse behavior test including open field test (OFT) and forced swimming test (FST) was evaluated before, 1 week, and 3 weeks after surgery. The behavior of the mice was scored according to the method described by (Brenes Sáenz et al., 2006). For OFT, the mice were placed in a 100 cm × l00 cm × 40 cm black open box with the bottom edge equally divided into nine compartments, and each mouse was placed in the middle compartment and timed for 5 min, and the total distance traveled by each mice was recorded. For FST, a transparent cylindrical glass tank of 18 cm in diameter and 20 cm in height was filled with warm water at 25°C to a depth of approximately 10 cm. The mice were allowed to float for 120 s without touching the bottom of the tank, and the duration of immobility was recorded in a total period of 4 min to assess the depression state. The immobility criteria were as follows: the mice had their heads above the water surface, without moving their limbs or were only slightly moving.

### 2.4 Plasma E2, T, and PRL enzyme-linked immunosorbent assay

Blood was collected into the procoagulant tube, and the serum was prepared for standby. The concentration was determined by ELISA. ELISA kit was provided by Jiangsu Jianglai Biotechnology Co. Ltd, Changsha, Hunan, China. Blood lipids in serum samples were determined by the automatic biochemical instrument, such as serum E2, T, and PRL. Regression equations for each sex hormone are the following: E2 (y= −0.445 + 107.539x+13.51, R^2^ = 0.998), T (y = −0.672x + 128.883 + 34.21, R^2^ = 0.998), PRL (y = 0.103 + 4. 611x + 14. 21, R^2^ = 0.998).

### 2.5 16S rRNA gene sequencing of the gut microbiome

The gut microbiome of each mice has been tested by 16S rRNA gene sequencing technique. The 16S rRNA gene is a conserved region within bacterial genomes, and the sequences of the 16S rRNA gene are highly specific to different bacteria, making it useful for identifying bacterial species. 16S rRNA gene sequencing involves PCR amplification of 16S rRNA gene fragments, followed by high-throughput sequencing, and finally bioinformatic analysis to compare the sequences with known sequences in databases to determine the bacterial species. Liquid chromatography-mass spectrometry (LC-MS) is an analytical chemistry technique that separates compounds in a sample using liquid chromatography and then detects and identifies these compounds using mass spectrometry. In microbiology, LC-MS can be used to analyze metabolites produced by bacteria, thereby indirectly identifying the bacterial species.

Bemac SMRT sequencing technology was used to accurately obtain full-length 16S rRNA gene sequences (Zhang et al., 2021). Total microbial genomic DNA from each sample was extracted using the MN NucleoSpin 96 So DNA extraction kit, following the manufacturer’s instructions. The extraction process involved several critical steps: microbial cells were lysed to release genomic DNA through mechanical disruption, chemical lysis with lysis buffer, or enzymatic digestion using proteinase K; proteins and other impurities were removed using phenol-chloroform extraction; potential inhibitors were filtered out to ensure DNA purity; DNA was then bound to a silica membrane; the membrane-bound DNA was washed to eliminate residual contaminants; and finally, the DNA was dried and eluted, resulting in a pure DNA solution ready for further analysis.

To amplify the V3–V4 region of the bacterial 16S rRNA gene, we used forward primer 338F (5′-ACTCCTACGGGGAGGCAGCA-3′) and reverse primer 806R (5′-GGACTACHVGGGTWTCTAAT-3′). The PCR amplification procedure included the following steps: the extracted DNA served as the template for the PCR reaction. The PCR reaction was performed in a thermal cycler with the following cycling conditions: denaturation at 94°C–98°C to separate the double-stranded DNA into single strands; annealing at 50°C–65°C to allow primers to bind to the single-stranded DNA templates; and extension at 72°C, where Taq DNA polymerase synthesized new DNA strands by extending from the primers.

The PCR products were then purified, quantified, and homogenized to form a sequencing library. Specific steps included purification of samples using OMEGA DNA purification columns to remove excess primers and other contaminants, assessment of purified PCR products using 1.8% agarose gel electrophoresis to confirm the presence and correct size of the DNA fragments, excision and recovery of suitable DNA fragments from the gel using the Monarch DNA Gel Recovery Kit to ensure high-purity DNA fragments, and preparation of the purified DNA for sequencing.

The final purified PCR products were sequenced using the Illumina NovaSeq 6000 sequencing platform. The sequencing process generated raw data, which underwent processing and analysis on the Illumina platform to produce high-quality and high-coverage gene sequence data. Additionally, SMRT sequencing technology and the PacBio Sequel platform were used for further analysis. These analyses were conducted at Bemac Personal Biotechnology Co., Ltd. in Beijing, China. By following these detailed procedures, we ensured the accuracy and reliability of our gut microbiome 16S rRNA gene sequencing, providing comprehensive insights into microbial composition and diversity.

### 2.6 Bioinformatics analysis

Sequences were clustered using USEARCH (version 10.0) software to cluster valid sequences into OTUs with 97% similarity, and representative sequences of OTUs were defined by taxonomy. Based on the taxonomy, the community structure was statistically analyzed at different levels of classification. For linear discriminant analysis effect sizes (LEfSe), the Kruskal–Wallis rank sum test and the Wilcoxon rank sum test were performed, and then, linear discriminate analysis (LDA) effect size charts and heatmaps were used to illustrate the differences in metabolites between groups. The KEGG database was used to perform pathway enrichment analysis of metabolites in difference.

### 2.7 Statistical analysis

All data are presented as mean ± standard deviation (*x* ± SD). Statistical comparisons between multiple treatment groups were performed using one-way ANOVA, repeated measures ANOVA, and Spearman correlation tests to identify the correlation between gut microbiome and metabolome. The graphs were constructed by R software (Version 3.5.0) and GraphPad Prism 9. Results with a *p*-value < 0.05 were considered to be statistically significant.

## 3 Results

### 3.1 Establishment of the OVX mouse model

The vaginal cytology of the CMD, CHM, and CYYNS (n = 6) groups was tested on days 1–6 to demonstrate successful diestrus phase post-OVX. The successful OVX was indicated by the presence of rare cornified squamous epithelial cells in the vaginal smear; however, leukocytes predominated in CMD, CHM, and CYYNS groups (McLean et al., 2012).

### 3.2 Behavioral experiments

#### 3.2.1 The result of OFT

**Figure 1:**
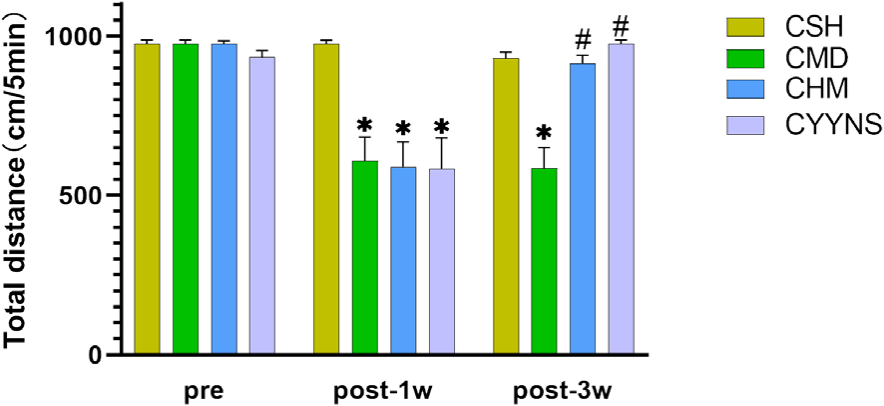
Histograms show the total distance difference of OFT between four groups in preoperative and 1 week and 3 weeks postoperative. (The OFT measures the total distance traveled by mice within a set time frame to assess their locomotor activity and depressive-like behavior. A reduction in distance indicates depressive-like behavior, which is alleviated after E2 and YYNS interventions. The values were expressed as mean ± standard deviation. Compared to the CSH group, *p<0.05; compared to CMD group, ^#^ *p*<0.05. The experiment was analyzed using repeated measures ANOVA to assess differences across time points, and it was repeated three times to ensure the reliability and reproducibility of the results. CSH, sham group (*n* = 6); CMD, the model group is the OVX without intervention (*n* = 6); CHM is the OVX model treated with E2 (*n* = 6); CYYNS is the OVX model treated with YYNS (*n* = 6).

OFT showed that the total distance was significantly reduced in the CMD, CHM, and CYYNS groups compared to the CSH group in week 1 postoperative (*p* < 0.05). It manifested that depression occurred after the OVX. In week 3 after OVX, the total distance in CMD was statistically significantly short compared to CSH, CHM, and CYYNS groups (*p* < 0.05). It manifested that after the administration of the intervention, depression was effectively alleviated in CHM and CYYNS 3 weeks after OVX (**Figure 1**).

#### 3.2.2 The result of FST

**Figure 2:**
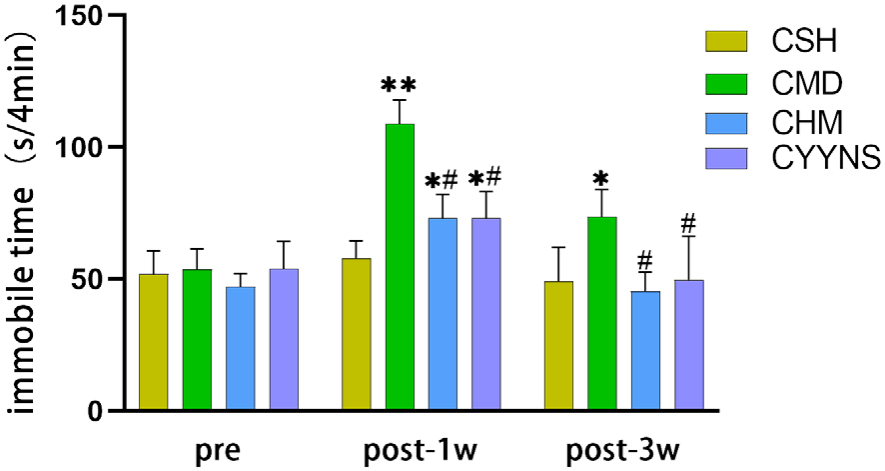
Histograms showing the immobile time of FST in preoperative and 1 week and 3 weeks postoperative. In FST measures, the longer periods of immobility indicate the more severe depressive-like behavior. Compared with the CSH group, *p<0.05; compared with the CMD group, ^#^ *p*<0.05. The experiment was analyzed using repeated measures ANOVA to assess differences across time points, and it was repeated three times to ensure the reliability and reproducibility of the results. CSH, sham group (*n* = 6); CMD, the model group is the OVX without intervention (*n* = 6); CHM is the OVX model treated with E2 (*n* = 6); CYYNS is the OVX model treated with YYNS (*n* = 6).

FST showed that 1 week after OVX, compared with the CSH group, the immobilization time of the CMD groups was significantly increased (*p*<0.01) and that of CHM and CYYNS groups (*p*<0.05). CHM and CYYNS have short immobilization compared with the CMD group (*p*<0.05). In week 3 post-OVX, the immobilization time of the CMD group was prolonged compared with the other groups (*p* < 0.05) (**Figure 2**).

### 3.3 ELISA result of E2, PRL, and T

**Figure 3:**
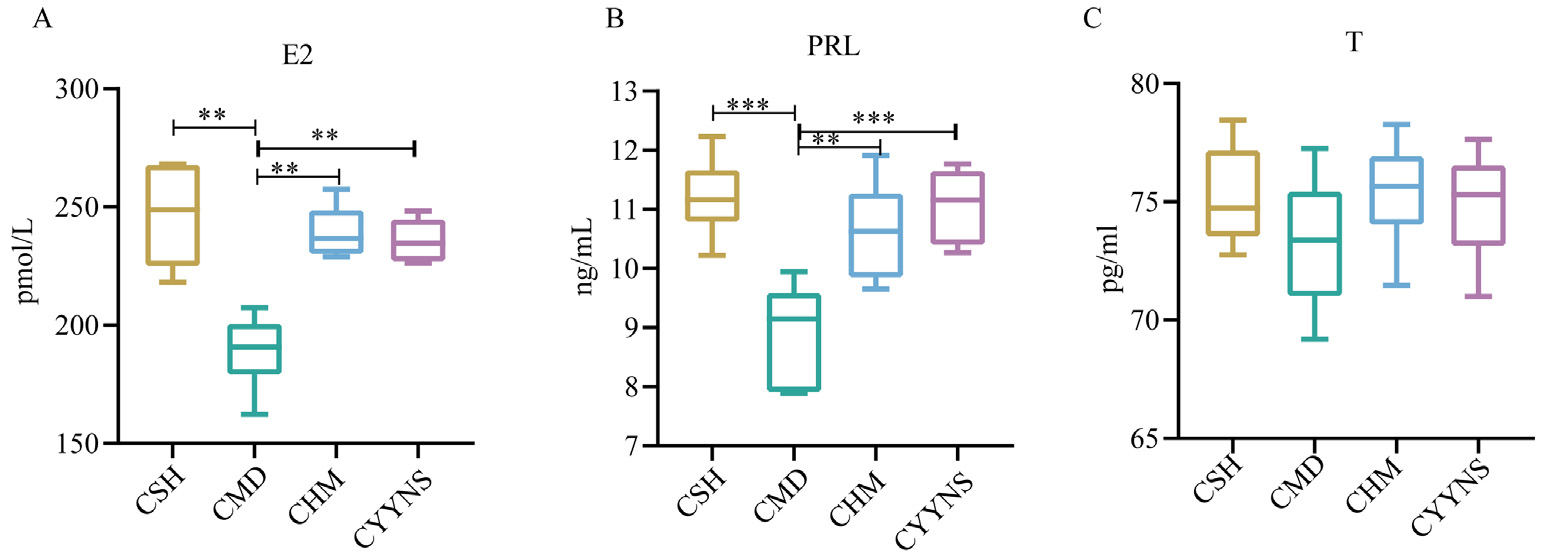
The boxplots show sex hormone difference comparation: **(A) **serum levels of E2, **(B)** serum levels of PRL, and **(C)** serum levels of T. The values were expressed as mean ± standard deviation. The experiment was analyzed using one-way ANOVA to assess differences in serum sex hormone level, and it was repeated three times to ensure the reliability and reproducibility of the results. Statistical significance: **p<0.01, ***p<0.01; CMD, the model group is the OVX without intervention (*n* = 6); CSH, sham group (*n* = 6); CHM is the OVX model treated with E2 (*n* = 6); CYYNS is the OVX model treated with YYNS (*n* = 6).

The E2 concentration in the CMD group was (189.05 ± 15.56) pmol/L, which was significantly lower than that in the CSH group (246.49 ± 23.36), the CHM group (239.32 ± 11.33), and the CYYNS group (235.78 ± 9.53) pmol/L (*p*<0.01). The CMD group has the lower PRL concentration (8.92 ± 0.85) ng/mL compared with the CSH group (11.20 ± 0.66), the CHM group (10.63 ± 0.83), and the CYYNS group (11.06 ± 0.66) ng/mL (*p*<0.01). There was no serum T concentration difference in CSH, CMD, CHM, and CYYNS groups. The serum T concentration in the four groups had no statistical significance (*p* > 0.05) (**Figure 3**).

### 3.3 Diversity of intestinal contents flora in mice

**Figure 4:**
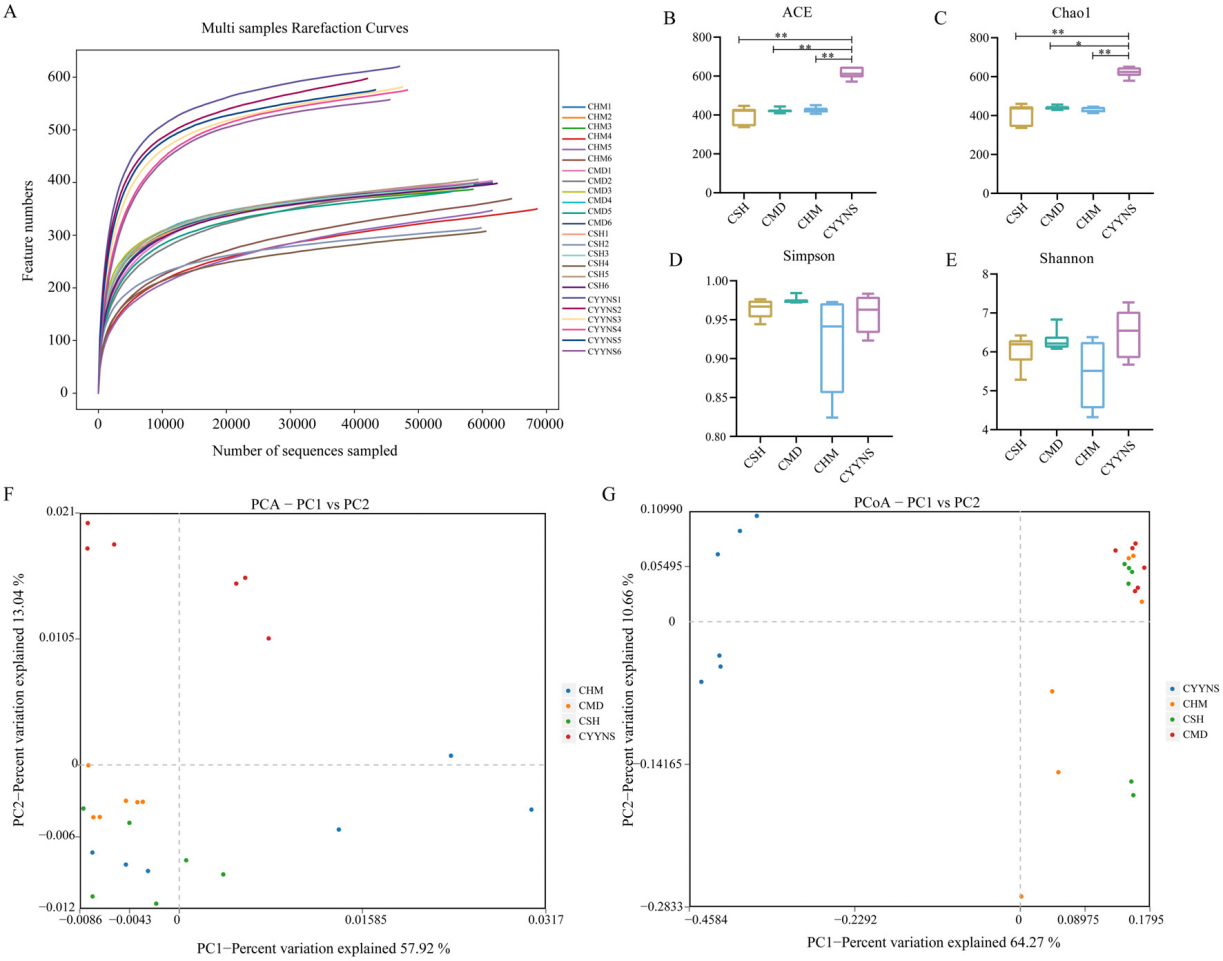
Box plots showing species diversity analysis in each group: **(A)** Species dilution curve, **(B)** ACE index, **(C)** Chao1 index, **(D)** Simpson index, **(E)** Shannon index, **(F)** principal component analysis, and **(G)** principal coordinates analysis. Data of ACE index, Chao1 index, Simpson index, and Shannon index were analyzed using one-way ANOVA to assess differences across time points, and it was repeated three times to ensure the reliability and reproducibility of the results. (Statistical significance: *p<0.05, **p<0.01; CMD, the model group is the OVX without intervention (*n* = 6); CSH, sham group (*n* = 6); CHM is the OVX model treated with E2 (*n* = 6); CYYNS is the OVX model treated with the YYNS (*n* = 6).

The dilution curve can be used to indicate whether the sample sequencing data are reasonable or not. Operational taxonomic units (OTUs) are used in ecological and microbiological studies to classify groups of closely related individuals based on genetic similarity, typically used in microbial ecology to represent species or operational groups for diversity analyses. **Figure 4A** shows the rarefaction curves for each sample, which plot the number of observed features (OTUs or ASVs) as a function of the number of sequences sampled. The curves plateau as the number of sequences increases, indica, indicating that the sequencing data can satisfy the current analysis. Each curve represents a sample, and different groups (CSH, CMD, CHM, and CYYNS) are color-coded. The flattening of the curves suggests that most diversity in the microbial communities has been captured at the current sequencing depth.

Alpha diversity reflects species abundance and species diversity of individual samples, where Chao1 and ACE indices measure species abundance, and Shannon and Simpson indices are used to measure species diversity. Chao1 is a metric used to estimate species richness, focusing particularly on the number of rare species—those observed only once or twice, which helps predict the total number of species that may be present in a community, especially when not all species are observed. The ACE index is similar to Chao1, also estimating species richness but emphasizing the abundance of rare species, making it valuable for assessing biodiversity and understanding community composition. As shown in **Figures 4B, C**, Chao1 and ACE indices were significantly higher in the CYYNS group compared with the CSH, CMD, and CHM groups (*p*<0.05).

Although the Shannon and Simpson indices did not show statistical significance (*p*<0.05), it can be observed from **Figures 4D, E** that the Simpson index of the CMD group is lower compared to the CSH, CHM, and CYYNS groups, while the Shannon index of the CYYNS group is higher compared to the CSH, CMD, and CHM groups. The Simpson index measures species diversity, with lower values indicating higher diversity and is used to assess evenness and stability in communities. The Shannon index also measures diversity by considering both richness and evenness, with higher values indicating greater diversity, commonly used to evaluate community stability. Our study further assessed beta diversity based on the Bray–Curtis distance algorithm, which mainly compares the object. As to the presence or absence of species, if the number of beta diversity of two populations is smaller, it shows that the two species are more similar, in which PCA and PCoA analyses take a down scaling approach to observe the differences between individuals or populations. As shown in **Figure 4F**, the scatter plot shows the results of PCA analysis, which reduces the complexity of the data by capturing the most important variations across the groups. The CMD group clusters separately from the CSH group, while CHM and CYYNS groups shift closer to CSH, indicating that treatment with E2 or YYNS modulates microbial community structure. The first two principal components (PC1 and PC2) explain 57.92% and 13.34% of the variance, respectively. As shown in **Figure 4G**, the scatter plot shows the PCoA results, which also capture the variance between the groups based on their microbial community composition. CMD clusters separately from the other groups, while the CHM and CYYNS groups are closer to the CSH group, indicating that both treatments have a significant effect on restoring the microbial community structure. The first two axes (PC1 and PC2) explain 64.27% and 10.65% of the total variance, respectively. The results showed that after Yangyin-ningshen formula treatment, the CYYNS group was farther away from the CSH and CMD groups, and the group differences were large (**Figure 4**).

### 3.4 Flora composition of mouse intestinal contents

**Figure 5:**
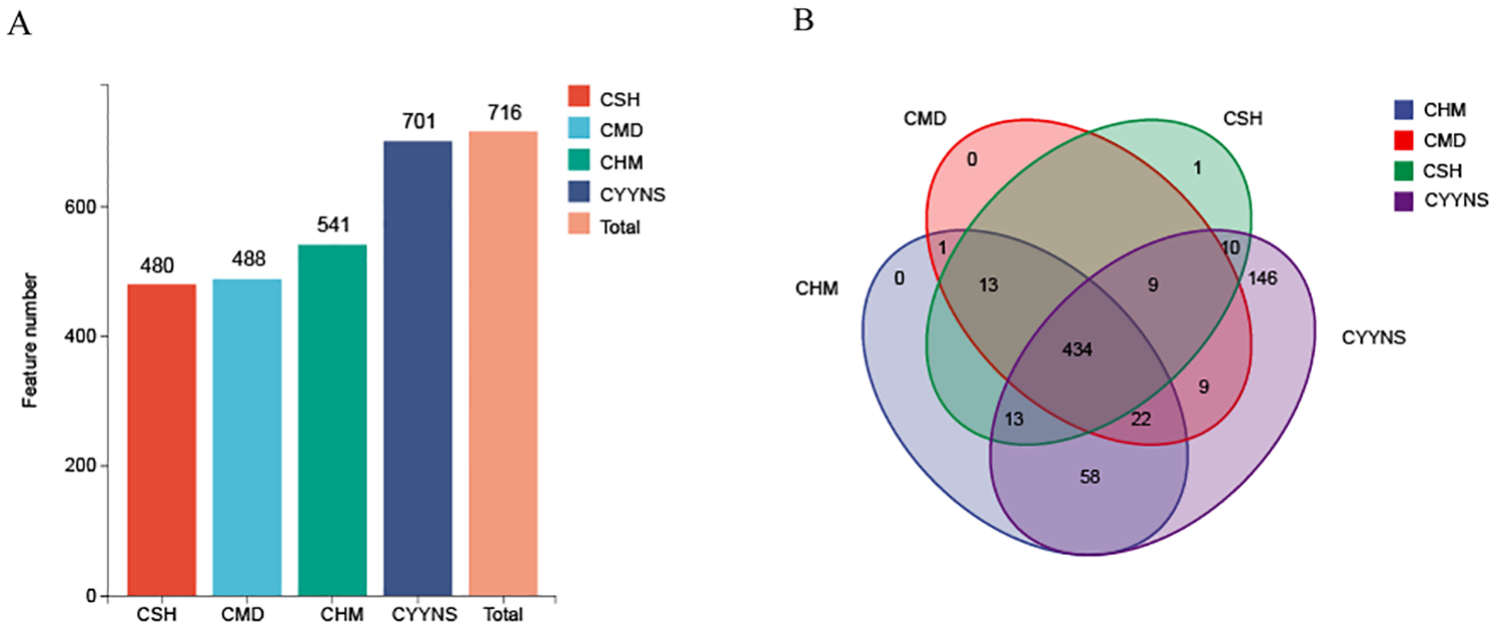
Histograms of OTU distribution in each group. **(A)** OTU number in each group, **(B)** Venn shows the same and different OTU. Model group is the OVX without intervention (*n* = 6); CSH, sham group (*n* = 6); CMD, CHM is the OVX model treated with E2 (*n* = 6); CYYNS is the OVX model treated with YYNS (*n* = 6).

As shown in **Figures 5**, sequences with higher than 97% similarity were grouped into one OTU cluster, with a total of 480 OTUs in the CSH group, 488 OTUs in the CMD group, 541 OTUs in the CHM group, and 701 OTUs in the CYNS group. These results suggest that treatment with CHM and CYYNS leads to an increase in microbial diversity, with the CYYNS group exhibiting the highest feature count. As shown in **Figure 5B**, the Venn diagram illustrates the number of shared and unique microbial features between the four groups. The core microbiome, represented by the 434 features shared by all four groups, forms the center of the diagram. Additionally, there are unique features for each group: the CYYNS group has 58 unique features, CHM has 13, CSH has 9, while CMD has no unique features. There are also smaller overlaps between the groups: 22 features are shared by CSH, CHM, and CYYNS, while 146 features are shared between CSH and CYYNS, indicating a greater similarity between these groups compared to CMD. The diagram emphasizes the unique microbial diversity observed in the CYYNS group, which shows the highest number of both shared and unique features.

**Figure 6:**
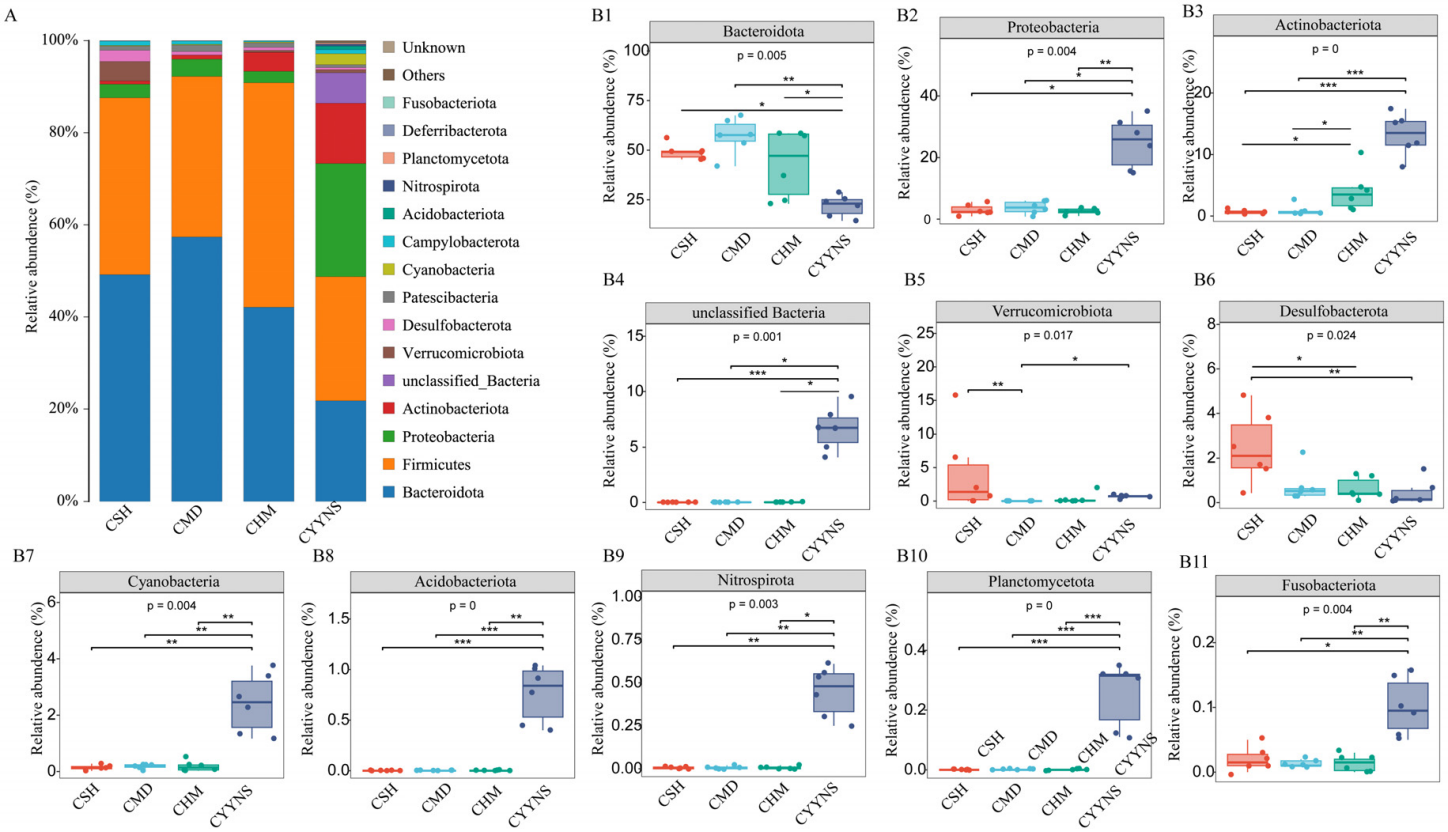
**(A)** Histograms of the relative abundance of colon flora at phylum level. **(B)** Box plots of the relative abundance of the microbiota of the colon flora at phylum level. The experiment was analyzed using one-way ANOVA to assess colon flora difference at phylum level, and it was repeated three times to ensure the reliability and reproducibility of the results. Statistical significance: *p<0.05, **p<0.01, ***p<0.00; CMD, the model group is the OVX without intervention (*n* = 6); CSH, sham group (*n* = 6); CHM is the OVX model treated with E2 (*n* = 6); CYYNS is the OVX model treated with the YYNS (*n* = 6).

To clarify which bacteria differ between the four groups, we analyzed the composition and variation of the top 15 gut microbiota relative abundances at the phylum level. As shown in **Figures 6A, B**, Bacteroidota, Firmicutes, Proteobacteria, and Actinobabacteria were the dominant microbiota of the mice colon flora in all groups. The relative abundance of Verrucomicrobiota was significantly lower in the CMD group compared to the CSH group (*p*<0.05). The relative abundance of Bacteroidota was significantly lower (*p*<0.05) in the CYYNS group compared to that in the CSH, CMD, and CHM groups, and the relative abundance of Proteobacteria, Actinobabacteria, *unclassified Bacteria*, Cyanobacteria, Acidobacteriota, Nitrospirota, Planctomycetota, and Fusobacteriota was significantly higher in the CYYNS group (*p*<0.05).

**Figure 7:**
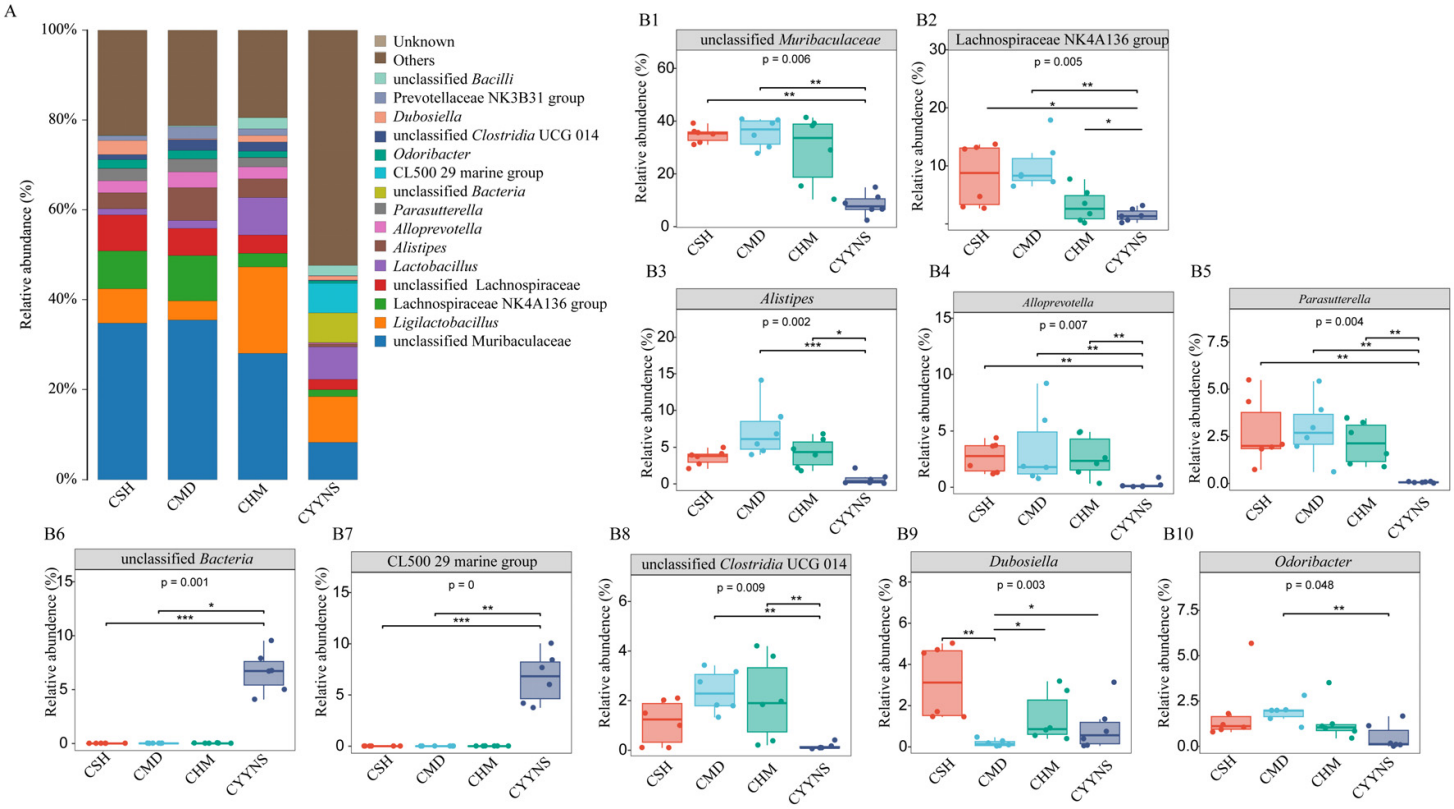
**(A)** Histogram of the relative abundance of intestinal contents at genus level. **(B)** Box plots of the relative abundance of the relative abundance of the microbiota of the intestinal contents at the genus level. The experiment was analyzed using one-way ANOVA to assess colon flora difference at the genus level, and it was repeated three times to ensure the reliability and reproducibility of the results. Statistical significance: *p<0.05, **p<0.01, ***p<0.001; CMD, the model group is the OVX without intervention (*n* = 6); CSH, sham group (*n* = 6); CHM is OVX model treated with E2 (*n* = 6); CYYNS is the OVX model treated with the YYNS (*n* = 6).

We further statistically analyzed the characteristic bacteria at the genus level. As shown in **Figures 7A, B**, the relative abundance of unclassified *Muribaculaceae*, *Alistipes*, *Alloprevotella*, and *unclassified Clostridia UCG 014* in the CMD group was significantly higher (*p*<0.05). The relative abundance of *Dubosiella* was significantly lower in the CMD group compared to the CSH group (*p*<0.001) and CHM and CYYNS groups (*p*<0.05). The relative abundance of *Odoribacter* was significantly lower in the CYYNS group compared to the CMD group (*p*<0.01). The relative abundance of *Lachnospiraceae NK4A136 group*, *unclassified Muribaculaceae*, *Alistipes*, *Alloprevotella*, *Parasutterella, CL500 29 Marine group*, and *unclassified Clostridium UCG 014* in the CYYNS group compared to the CSH group, CMD group, and CHM group was significantly lower (*p*<0.05), while that in the *CL500 29 Marine group* and *unclassified bacteria* was significantly higher (*p*<0.05).

### 3.5 Microbiota characterizing intestinal contents flora in mice

**Figure 8:**
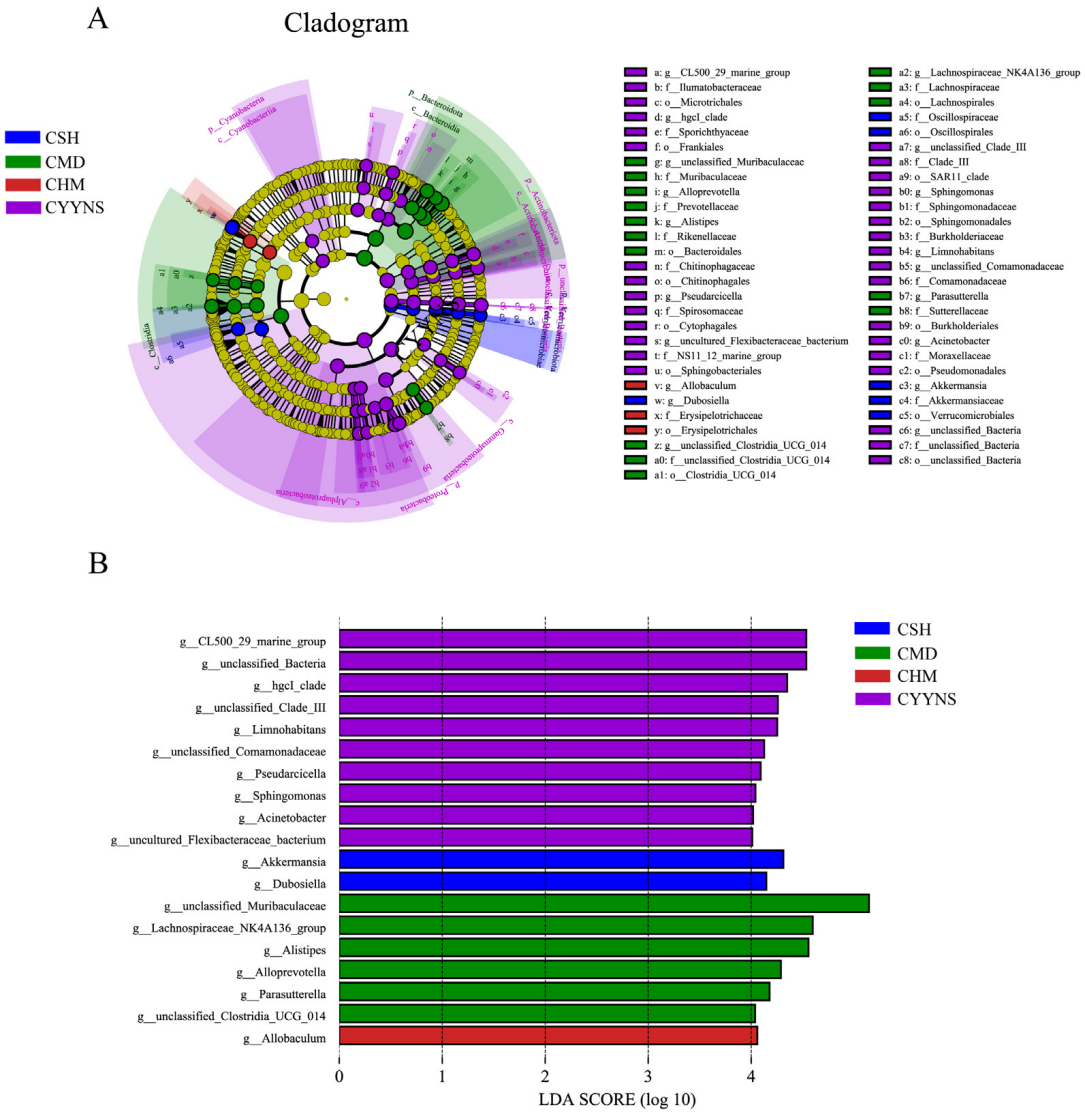
**(A)** The clade diagram generated by LEfSe analysis showed the phylogenetic distribution of intestinal contents from phylum to species, **(B)** the histogram of the distribution of LDA values at the genus level to identify different groups of bacterial species. The statistical method is linear discriminant analysis. CMD, the model group is the OVX without intervention (*n* = 6); CSH, sham group (*n* = 6); CHM is OVX model treated with E2 (*n* = 6); CYYNS is the OVX model treated with YYNS (*n* = 6).

In order to compare abundance difference in species between groups at a given taxonomic level, we identified species with significant differences between groups based on a linear discriminant analysis of LEfSe. Differences in microbiota abundance were observed in the CSH, CMD, CHM, and CYYNS groups, with multiple bacteria identified as key discriminators. LEfSe analysis of the evolutionary clodogram circles radiating from inside to outside represent taxonomic levels from phylum to genus. As shown in **Figure 8A**, there were significantly different species in each group, with the CYYNS group of unclassified Bacteria being significantly different from the phylum level to the genus level. As shown in **Figure 8B**, the length of the histogram of the distribution of LDA values represents the magnitude of the effect of the differing species, with 19 bacteria significantly enriched at the genus level when the LDA score was >4. The CSH group was significantly enriched in *Akkermansia* and *Dubosiella*. The CMD group had six significantly different species, with *unclassified Muribaculaceae* scoring the highest. There were 10 significantly different species in the CYYNS group, with larger LDA scores in the unclassified Bacteria and *CL500 29 marine groups*, whereas only the CHM group was significantly enriched in *Allobaculum.*

### 3.6 Functional gene prediction of mouse intestinal content flora

**Figure 9:**
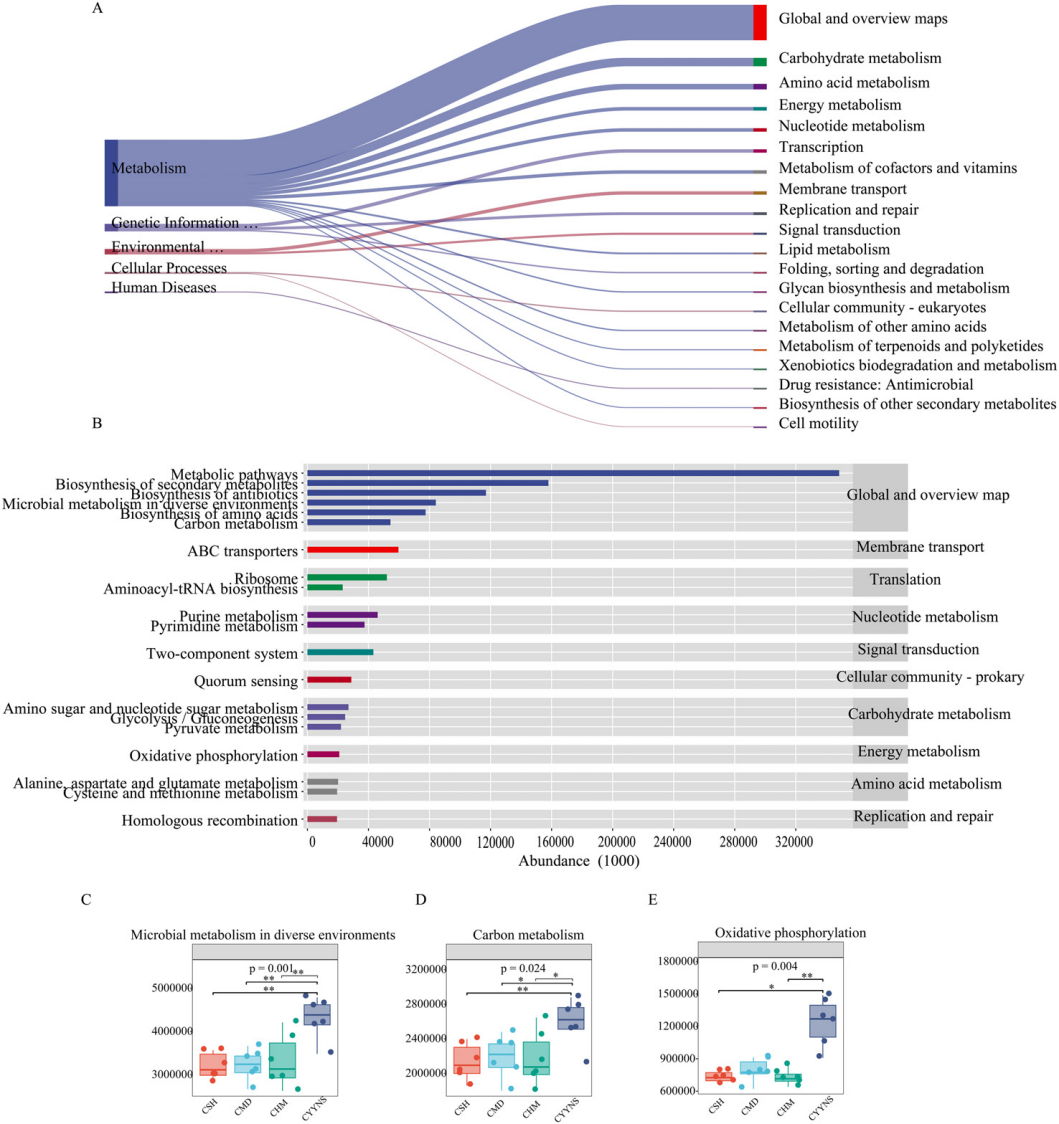
**(A)** Sankey diagram of primary and secondary metabolic pathways: the left side is the primary metabolic pathway, the right side is the secondary metabolic pathway, the direction of the line, that is, the direction of the data flow, and the width of the line represents the magnitude of the abundance. **(B)** The horizontal distribution histogram: the abscissa is the abundance of the metabolic pathway, the ordinate is the tertiary classification of the metabolic pathway, the rightmost is the secondary classification to which the pathway belongs, and **(C)** microbial metabolism in diverse environment abundance plot, **(D)** carbon metabolism abundance plot, and **(E)** oxidative phosphorylation abundance plot. The experiment was repeated three times, and data were analyzed using one-way ANOVA to assess the difference of microbial metabolism in diverse environments abundance, carbon metabolism abundance, and oxidative phosphorylation abundance at metabolism level. Statistical significance: *p<0.05, **p<0.01; CMD, the model group is the OVX without intervention (*n* = 6); CSH, sham group (*n* = 6); CHM is OVX model treated with E2 (*n* = 6); CYYNS is the OVX model treated with YYNS (*n* = 6).

To determine the metabolic and functional effects of CYYNS on the gut flora, microbiota-associated metabolic pathways were predicted by PICRUSt2 analysis based on the KEGG database. Level 1 metabolic functional genes are mainly composed of cellular processes, environmental information processing, genetic information processing, human diseases, glycan pathways, and metabolism functional genes, of which the metabolism pathway had the highest abundance. As shown in **Figure 9**, we screened the top 20 secondary metabolic pathways in terms of abundance, mainly consisting of global and overview maps, carbohydrate metabolism, amino acid metabolism, energy metabolism, nucleotide metabolism, and transcription, with the global and overview maps pathway having the greatest abundance (**Figure 9A**). The top 20 tertiary metabolic pathways in terms of abundance were selected, mainly biosynthesis of secondary metabolites and antibiotics, microbial metabolism in diverse environments, and biosynthesis of amino acids (**Figure 9B**). Microbial metabolism in diverse environments and carbon metabolism abundance were significantly higher in the CYYNS group compared to the CSH, CMD, and CHM groups, and oxidative phosphorylation abundance was significantly higher in the CYYNS group compared to the CSH and CHM groups (*p* < 0.05, **Figures 9C–E**).

### 3.7 Correlation analysis between hormones and gut flora

**Figure 10:**
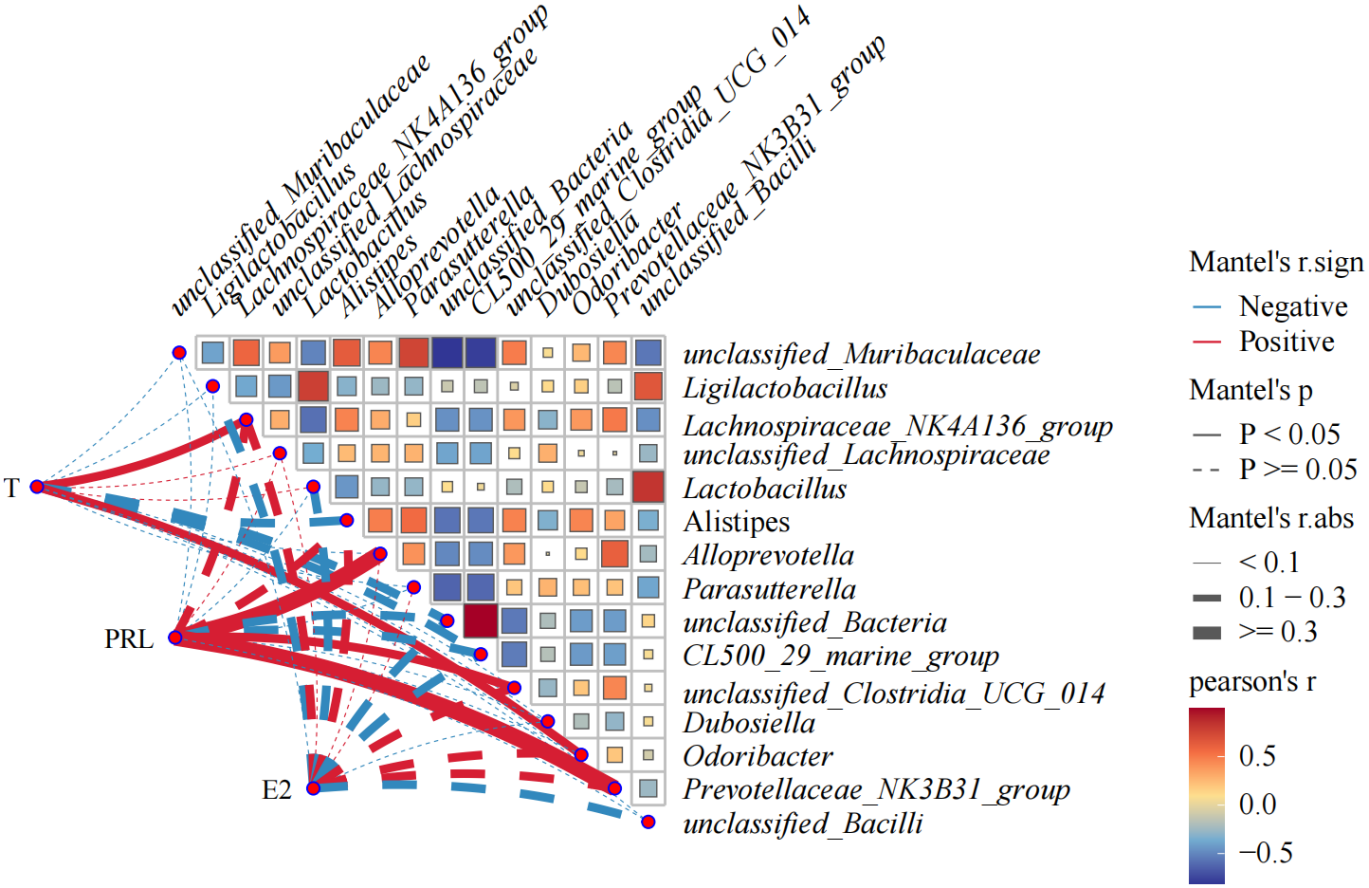
Heat map showing correlation analysis between E2, PRL, T, and gut flora, and correlation analysis among gut flora. The statistical method is Pearson’s correlation analysis. Pearson’s R>0.0 color red indicates positive correlation; Pearson’s R<0.0 color blue indicates negative correlation. The darker the color is, the more the correlation will be. CMD, the model group is the OVX without intervention (*n* = 6); CSH, sham group (*n* = 6); CHM is OVX model treated with E2 (*n* = 6); CYYNS is the OVX model treated with YYNS (*n* = 6).

In order to further study the correlation between flora change and hormone level in OVX mice, bacteria with the top 15 relative abundance of intestinal flora were selected for Pearson’s correlation analysis with T, E2, and PRL. The correlation network plot in **Figure 10** showed that T was significantly positively correlated with *Lachnospiraceae NK4A136 group* and *Odoribacter* (*p*<0.05). PRL was significantly and positively correlated with *Alloprevotella*, unclassified *Clostridia UCG 014*, and *Prevotellaceae NK3B31* group (*p*<0.05). The correlation heat map indicated that unclassified Bacteria had an intensive positive correlation with *CL500 29* marine group (R=0.99) and a strong negative correlation with *unclassified Muribaculaceae* (R= 0.81). *Unclassified Muribaculaceae* showed strong negative correlation with *CL500 29 marine* group (R=0.78) and *Parasutterella* (R=0.74). *Unclassified Bacilli* showed positive correlation with *Lactobacillus* intensively (R=0.83).

## 4 Discussion

Previous studies suggested that PDD is a prevailing psychiatric condition induced by abrupt sex hormone decline especially E2, which needs more attention. A study of Women’s Health Across the Nation (SWAN) found that the prevalence of depression in women at different stages of the perimenopausal period was 20.9% at baseline, 27.8% in the early perimenopausal transition, stabilized at 25.2% in the late perimenopausal transition, and decreased to 22% by post-menopause (Bromberger et al., 2011). The prevalence of depression was even higher in Zhejiang, China, where the prevalence of perimenopausal depression was 47.43%, with the majority of women experiencing mild (38.56%) or moderate depressive symptoms (8.00%) and 0.86% experiencing severe depressive symptoms (Chu et al., 2022). Stress, smoking, obesity, and educational attainment have all been associated with increased reports of depression (Bromberger et al., 2011). Estrogen is one of the most important sex hormones secreted by the ovaries, and estrogen is not only associated with the occurrence of PDD, but estrogen levels are strongly associated with peri-menopausal symptoms (Chu et al., 2022). The decrease in estrogen levels during peri-menopause leads to the increase risk of PDD (Org et al., 2016), in addition to a decrease in vaginal pH and *Lactobacillus* abundance (Shen et al., 2022).

E2 regulates the gut flora; a reduction in E2 levels can lead to gut microbiome disorders, characterized by a decrease in both the abundance and diversity of the gut microbiome (Shu et al., 2023). Colonic microbiota is a key focus of our microbial research, and it has a stronger correlation with perimenopausal depression. The colonic microbiota is intricately involved in the reabsorption of estrogen, whereas the microbiota of the small intestine cannot accurately reflect the correlation with estrogen (Baker et al., 2017). Furthermore, the chemical reactions within the colon differ from those outside the intestinal tract. Hence, we opt for colonic contents to study the correlation between gut microbiota and depression (Shu et al., 2023). Our previous research has also confirmed the distinction between intestinal fecal microbiota and intestinal mucosal microbiota (Qiao et al., 2024). Therefore, in the context of perimenopausal depression, we only consider changes in intestinal fecal microbiota and not mucosal changes (Guo et al., 2024).

This can have significant implications for overall health and well-being (Maeng and Beumer, 2023). Here are some specific details. First, E2 regulates the abundance of the gut microbiome. Our study found that the OUT numbers, Chao1, and ACE indices decreased post-OVX in individuals with low serum E2, indicating changes in gut microbiome abundance following a reduction in serum E2 levels. Second, E2 also regulates the composition of the gut microbiome. In healthy female individuals, a lower Firmicutes/Bacteroidetes ratio is typically observed. However, a reduction in E2 levels leads to an increase in the Firmicutes/Bacteroidetes ratio (Baker et al., 2017). Lastly, E2 modifies the gut microbiome through its metabolic activity, specifically by promoting the production of short-chain fatty acids (SCFAs) such as butyrate in the intestine. These SCFAs are essential for maintaining the integrity of the epithelial barrier. A low level of E2 can result in a reduction in SCFAs (Zeibich et al., 2021). SCFAs are crucial for the healthy colon epithelial cells. Consequently, a deficiency in SCFAs can damage the barrier integrity, which can lead to bacterial translocation, thereby exacerbating or inducing certain inflammatory diseases (Rivera and Bethea, 2013). Furthermore, E2 is beneficial in combating insulin resistance, which is susceptible to the adverse effects of obesity and gut microbiome disorders (Zeibich et al., 2021).

In our study, we found that serum E2 reduction in OVX mice led to a decrease in both the abundance and diversity of the gut microbiome and possessed low PRL level as well. With low serum E2, the number of OUT, Chao1, and ACE index decreased in CMD post-OVX. Besides gut microbiome alteration, low E2 led to the onset of depression notably. PRL is a hormone secreted by the pituitary gland; it does not directly affect depression (Qin et al., 2015). However, it influences E2 through HPO axis. When exogenous E2 increases, the HPO axis responds with a negative feedback mechanism, resulting in an increase in pituitary PRL secretion. The negative feedback of the HPO axis serves to counteract excessive estrogen levels (Qin et al., 2015). Therefore, we analyze that PRL elevation is not caused by estradiol’s influence but rather by the negative feedback effect of the HPO axis.

With E2 and YYNS intervention, depression symptom could alleviate effectively in mice post-OVX, as shown by the shortened immobilization time in OFT and FST post-OVX. Following E2 and YYNS intervention, serum E2 levels and PRL levels increased significantly.

Reproductive hormones play a significant role in the emotional regulation within the central nervous system. HPO refers to a complete and coordinated neuroendocrine system composed of the hypothalamus, pituitary gland, and ovary, which has an important influence on hormone secretion level, neuroendocrine function, and target organ function of the body, and it is closely related to the development of PDD (Amiel Castro et al., 2021). YYNS has the ability to elevate serum E2 levels. The effect is similar to supplementing exogenous E2. E2 controls hormone secretion from the hypothalamus and pituitary through a negative feedback mechanism, maintaining the balance of FSH and LH (Baker et al., 2017). When E2 levels decrease, this regulatory mechanism is disrupted, resulting in abnormal secretion of GnRH, FSH, and LH, which impairs follicle development and ovulation (Qin et al., 2015). These reproductive dysfunctions not only affect fertility but also trigger neurotransmitter imbalances, particularly in systems related to mood regulation (Parry, 2021). The reduction in E2 weakens the expression of BDNF and PSD-95, affecting synaptic plasticity and leading to emotional disturbances and depression (Jett et al., 2022). The results of the study also support the view that reproductive system dysfunction resulting from E2 and PRL depletion can lead to depression. Exogenous estrogen supplementation with E2 and YYNS increased plasma E2 level and effectively alleviated depression. This further demonstrate that the alleviation of depression by YYNS is closely related to the HPO axis.

Remarkably, apart from brain-behavioral changes after OVX in mice, chronic stress leads to dramatic gut microbial alteration, further illustrating complex and comprehensive E2–gut–brain axis regulation. Changes in estrogen levels can significantly alter the gut microbiota (Yatsonsky et al., 2019). Postmenopausal estrogen decline is associated with a reduced relative abundance of beneficial bacteria such as *Bifidobacterium* within the gut microbiota (Guan et al., 2023). This results in a significant decrease in the ratio of *Bifidobacterium* to Enterobacteriaceae, a reduction in Proteobacteria compared to premenopausal status, and lower relative abundances of *{Citation}Prevotella*, *Parabacteroides*, and *Bilophila* (Yatsonsky et al., 2019). Prospective clinical studies have found that the severity of hot flashes in postmenopausal women is positively correlated with *Porphyromonas* and *Prevotella corporis*, while *Clostridium asparagiforme* is associated with reduced hot flashes (Kahleova et al., 2023). Animal experiments have also shown a decrease in *Bacteroides*, *Bifidobacterium*, *Lactobacillus*, and *Akkermansia* abundance in postmenopausal rats (Fang et al., 2021). In postmenopausal women, the gut microbiota exhibits a higher Firmicutes/*Bacteroides* ratio, with reduced *Bacteroides* abundance and increased relative abundances of *Lachnospira* and *Roseburia* (Yatsonsky et al., 2019).

Decreased E2 leads to a significant reduction in both diversity and abundance of the mouse microbiota post-OVX. Our study observed that following YYNS intervention, the relative abundance of *Bacteroidetes* decreased at the phylum level, while those of Proteobacteria, Actinobacteria, unclassified Bacteria, Cyanobacteria, Acidobacteriota, Nitrospirota, Planctomycetota, and Fusobacteriota significantly increased. Exogenous supplement of E2 can improve the diversity and abundance of intestinal flora. YYNS has the highest potency to increase the diversity and abundance of intestinal flora. At the genus level, the abundances of *Lachnospiraceae NK4A136 group*, *unclassified Muribaculaceae*, *Alistipes*, *Alloprevotella*, *Parasutterella*, *Dubosiella*, *Odoribacter*, and *unclassified Clostridium UCG 014* were significantly reduced with YYNS intervention. Conversely, YYNS increased the abundances of *CL500-29 marine* group and *unclassified Bacteria.* Notably, the abundance of *unclassified Bacteria* was significantly elevated from the phylum level to the genus level.

Followed by dramatic E2 changes, the integrity disruption of HPO results in gut flora disorder via the E2–gut–brain axis. After OVX, relative abundance levels of Verrucomicrobiota, Proteobacteria, Actinobacteria, Planctomycetota, Cyanobacteria, Acidobacteia, Actinobacteria, Nitrospirota, and Fusobacteria were significantly lower; on the contrary, the relative abundance levels of Bacteroidota and Desulfobacterota were significantly higher. Bacteroidota is a Gram-negative bacterial phylum. The cell wall is mainly composed of lipopolysaccharide (LPS). It prominently upgrades gut inflammation, depresses host innate immunity, and suppresses Bacteroidota, which can downgrade the inflammatory response in the gut (Li et al., 2023). Actinobacteria is one of the four phyla of gut microbiota, which plays a key role in the maintenance of intestinal homeostasis. Bifidobacteria, which belong to this phylum, is now widely used as a probiotic, capable of attenuating rapid blood glucose level and elaborating positive effects on host health (Abuqwider et al., 2023). It suggests that YYNS possesses anti-inflammatory and bacteria regulation property.

At the genus level, the abundances of *Lachnospiraceae NK4A136 group*, unclassified Muribaculaceae, *Alistipes*, *Alloprevotella*, *Parasutterella*, *Dubosiella*, *Odoribacter*, and *unclassified Clostridium UCG 014* were significantly reduced with YYNS intervention. Conversely, YYNS increased the abundances of CL500-29 marine group and *unclassified Bacteria*. Notably, the abundance of *unclassified Bacteria* was significantly elevated from the phylum level to the genus level.

*Dubosiella* has been linked to inflammation and immune responses (Cryan et al., 2019), while *Odoribacter* is a conditional pathogenic bacterium, which is positively relevant to the development of inflammation (Rivera and Bethea, 2013); restrain of *Odoribacter* abundance can effectively improve inflammation and symptoms in Parkinson’s disease (Du et al., 2022). Previous studies considered *Lachnospiraceae* and *Alloprevotella* as probiotics, but recent research has revealed that besides producing short-chain fatty acids and converting primary bile acids into secondary bile acids, *Lachnospiraceae* promotes inflammatory reactions through the synthesis of proinflammatory glucan polysaccharides, which can even trigger Crohn’s disease (Sorbara et al., 2020). Postmenopausal women exhibit increased relative abundance of *Lachnospiraceae*, which is closely associated with postmenopausal osteoporosis (Yatsonsky et al., 2019). *Alloprevotella* has been found to increase the risk of sepsis (Chen et al., 2023) and promote epithelial inflammatory responses, such as increasing the risk of oral squamous cell carcinoma (Zhang et al., 2020).

*Alistipes* are strongly associated with depression (Parker et al., 2020); they can be isolated in abscesses from the appendix and brain (Shkoporov et al., 2015). To be a Gram-negative microorganism, *Alistipes* abundance is positively correlated with inflammatory markers (Zhang et al., 2023). After 3 weeks intervention, by significantly reducing the relative abundances of *Lachnospiraceae NK4A136*, *unclassified Muribaculaceae*, *Alistipes*, *Alloprevotella*, *Parasutterella*, *Dubosiella*, *Odoribacter*, and *unclassified Clostridium UCG 014*, YYNS increases the relative abundance of *CL500-29 marine* and *unclassified Bacteria* at the same time inhibits inflammatory reactions, thereby improving depressive mood post-OVX.

Hormones were strongly correlated with gut flora (Yan et al., 2024); T was significantly positively correlated with *Lachnospiraceae NK4A136* and *Odoribacter* group. PRL was significantly positively correlated with *Alloprevotella*, *unclassified Clostridia UCG 014*, and *Prevotellaceae NK3B31* group showed significant positive correlation. Bacteria were also closely related to each other, with *unclassified Bacteria* showing a very strong positive correlation with *CL500 29 marine* group and a very strong negative correlation with *unclassified Muribaculaceae*; *unclassified Muribaculaceae* showing a very strong negative correlation with *CL500 29 marine* group and *Parasutterella*; and *unclassified Bacilli* showing a very strong positive correlation with *Lactobacillus*.

## 5 Conclusion

Our results illustrate that mice with OVX lead to depression behavioral change in addition to acute fluctuation in E2 and PRL levels, together with the occurrence of alterations in the diversity and abundance of the gut flora occur. E2 and YYNS intervention can both improve depression disorder, and the diversity and abundance of gut flora. In our study, the tri-directional dialog between E2, cerebral behavior, and gut flora revealing the E2–gut–brain axis by the regulation of cerebral function, HPO axis, and gut flora plays an important role in PDD. By elevating serum E2 level, YYNS modulated brain neuronal function to improve depressive behavior and acted on the gut microenvironment as endogenous E2. In summary, we hypothesize that YYNS effectively alleviates PDD that may be associated with the E2–gut–brain axis.

## Data Availability

The original contributions presented in the study are publicly available. This data can be found here: NCBI: PRJNA1031711 (https://www.ncbi.nlm.nih.gov/sra/PRJNA1031711).
